# Ontological Problem-Solving Framework for Dynamically Configuring Sensor Systems and Algorithms

**DOI:** 10.3390/s110303177

**Published:** 2011-03-15

**Authors:** Joseph Qualls, David J. Russomanno

**Affiliations:** 1 Department of Electrical and Computer Engineering, Herff College of Engineering, University of Memphis, 3720 Alumni Avenue, Memphis, TN 38152, USA; 2 Department of Electrical and Computer Engineering, Purdue School of Engineering and Technology, Indiana University-Purdue University Indianapolis (IUPUI), 799 W. Michigan St., Indianapolis, IN 46202, USA; E-Mail: drussoma@iupui.edu

**Keywords:** sensor networks, sensor ontology, profiling sensors, ontological framework

## Abstract

The deployment of ubiquitous sensor systems and algorithms has led to many challenges, such as matching sensor systems to compatible algorithms which are capable of satisfying a task. Compounding the challenges is the lack of the requisite knowledge models needed to discover sensors and algorithms and to subsequently integrate their capabilities to satisfy a specific task. A novel ontological problem-solving framework has been designed to match sensors to compatible algorithms to form synthesized systems, which are capable of satisfying a task and then assigning the synthesized systems to high-level missions. The approach designed for the ontological problem-solving framework has been instantiated in the context of a persistence surveillance prototype environment, which includes profiling sensor systems and algorithms to demonstrate proof-of-concept principles. Even though the problem-solving approach was instantiated with profiling sensor systems and algorithms, the ontological framework may be useful with other heterogeneous sensing-system environments.

## 1. Introduction

Dynamically matching sensor systems to algorithms to satisfy a task poses a significant challenge in sensor networks. The challenge is made even more difficult because sensor systems and algorithms are not typically designed independently, which often limits their reuse in tasks that may not have been anticipated when the sensors and algorithms were first deployed. Compounding the challenge is the lack of knowledge and data models, which describe sensor and algorithm capabilities, properties, and relationships [1–6]. The focus of this paper is on the reasoning process used in a novel ontological problem-solving framework, which can be leveraged by software agents on sensor networks, to opportunistically match sensor systems to independently designed algorithms to form synthesized systems capable of satisfying a task.

### 1.1. Ontological Problem-Solving Framework

**Figure 1.**
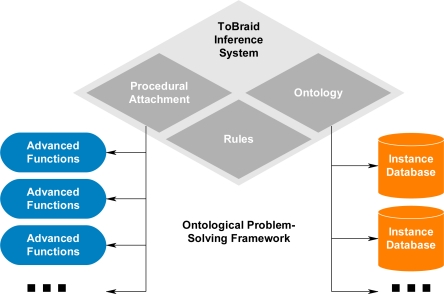
Overview of ontological problem-solving framework.

**Figure 2.**
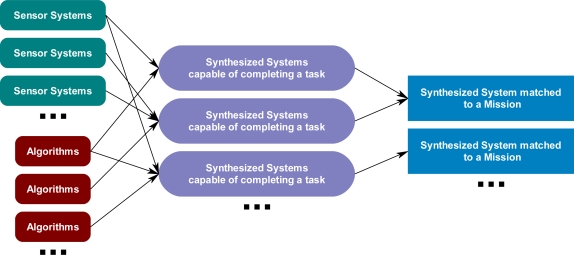
Creation of synthesized systems which are then assigned to subtasks of high-level missions via the ontological problem-solving framework.

The ontological problem-solving framework (Figure 1) has the overall goal to discover and match sensor systems to compatible algorithms to form a synthesized system, which is capable of satisfying a given subtask. The synthesized systems and other algorithms may then be matched to form more complex synthesized systems, which may then be assigned to tasks of high-level missions (Figure 2). The ontological problem-solving framework will then coordinate all matched and synthesized sensor systems and algorithms to complete the missions. The problem-solving approach could have been developed with standard database technologies and SQL queries. However, one of the issues that makes discovering and matching sensors to algorithms problematic is the lack of knowledge models used to describe those systems.

The knowledge models also need to leverage well-defined semantics in a machine-interpretable format so other agents may interact with the described systems. The requirement to opportunistically match sensors to algorithms increased the need to use ontologies (which specify the semantics) and rules based on description logic to infer which components may be used to form synthesized systems. The knowledge models used by the ontological problem-solving framework may then be leveraged by other systems for more complex inference if needed. The ontological problem-solving framework was developed using the TopBraid Maestro software by TopQuardrant [7], which uses the web ontology language (OWL) [1–6] for knowledge capture, SPARQL [8] for specifying rules, and the TopSpin inference engine for interpreting the rules. Other systems, such as Protégé, which uses JESS and SWRL [1–6], could have also been used to develop the ontological problem-solving framework. The main focus of this paper is to detail the reasoning process the ontological problem-solving framework uses to match sensor systems to compatible algorithms to form synthesized systems, which are capable of satisfying a given task.

### 1.2. Matching Sensors to Algorithms

**Figure 3.**
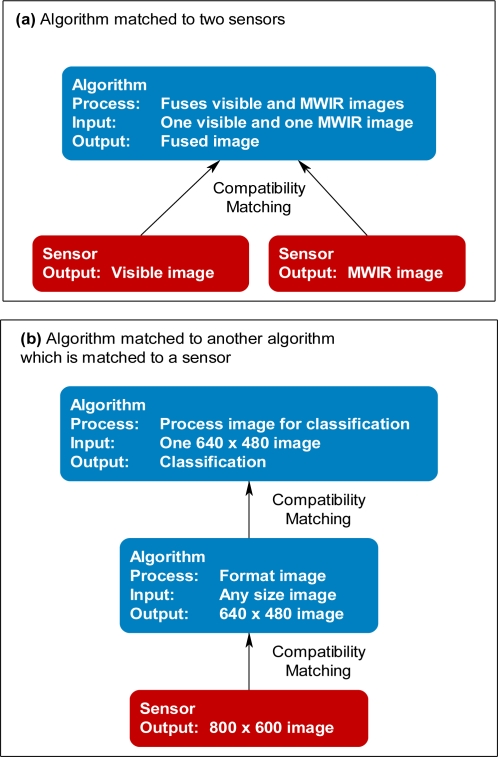
**(a)** Algorithm, which requires data from two sensor systems, matched to two compatible sensor systems. **(b)** Algorithm matched to a compatible algorithm, which is also matched to a compatible sensor system.

Engineers often design an algorithm for a specific sensor system. This dependence makes the algorithm difficult to use with other sensors opportunistically based on ever-changing persistence surveillance goals. If sensors and algorithms are designed independently, then, a problem-solving approach must enable the matching of a sensor to a compatible algorithm to achieve a task, such as formatting the sensor data for a specific purpose or extracting pixels from an imaging sensor for subsequent processing. The composition of matched sensor systems and compatible algorithms to achieve a task can be made even more difficult if an algorithm requires multiple data sources (Figure 3(a)), or if a chain of multiple sensors and algorithms must be composed to achieve subtasks supporting an overall task (Figure 3(b)). The problem-solving approach must describe the relationship between the preconditions and post conditions of the algorithms, as well as descriptions of the raw data, and possibly features generated by the sensor systems [9–12].

### 1.3. Related Work

**Figure 4.**
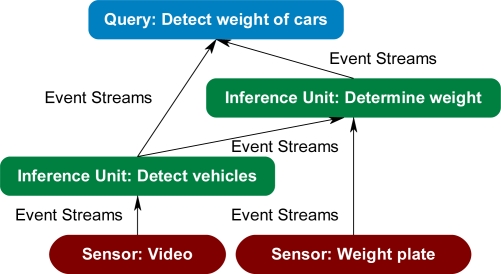
Semantic Streams query.

**Figure 5.**
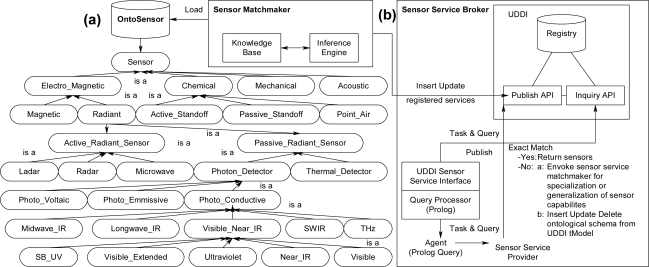
**(a)** Excerpt of the OntoSensor ontology. **(b)** Problem-solving for discovering and tasking sensor systems using OntoSensor.

There have been several approaches and tools developed to address in part the challenge of matching sensors to compatible algorithms. These techniques and tools include, but are not limited to, Sensor Fabric [9,13–15], Sensor OASiS [16], Agilla [17–19], Semantic Streams and SONGS [20,21], and CIEDETS [22,23]. Other research efforts focused on the development of ontologies that describe sensors and their respective capabilities, such as OntoSensor [2–6], Sensor Network Data Ontology [24], Sensor and Data Wrapping Ontology [25], Stimulus-Sensor-Observation Ontology [26], Sensor Observation and Measurement Ontology [27], Semantic Sensor Network Ontology [28], Disaster Management Sensor Ontology [29], and a survey of sensor ontologies [30] are also efforts relevant to our work. Other work promotes a logical model to follow while developing a problem-solving approach. For example, Sensor Modeling Language (SensorML) [31] describes high-level conceptual models using Unified Modeling Language (UML) of sensors, algorithms, and supporting notions to facilitate interoperability. The Open Geospatial Consortium (OGC) specify draft interoperability interface standards and metadata encodings that integrate sensor systems into information infrastructures, such as Observations and Measurements (O&M) [32,33], SensorML [34], Transducer Model Language (TML) [35], Sensor Observation Service (SOS) [36], Sensor Planning Service (SPS) [37], Sensor Alert Service (SAS) [38], and Web Notification Services (WNS) [39]. Semantic Streams and OntoSensor are two important efforts because of their use of semantics and ontologies. Semantic Streams and the follow up SONGS effort were developed by Microsoft to facilitate queries to determine capabilities and subsequent tasking of sensors and algorithms. Semantic Streams uses event streams, which are collected raw data from sensor systems with meta information attached, and inference units, which operate on event streams by creating semantic information about the event streams. Queries posted to Semantic Streams are broken down into one or more of the inference units (Figure 4). SONGS adds the use of an ontology to describe the inference units. Instead of queries being directly mapped to inference units, the approach can infer which inference units may satisfy a given query [20,21]. OntoSensor is a semantic-web-compatible ontology that captures knowledge about sensor systems (Figure 5(a)). OntoSensor can be used to create relationships to other sensor instances and to derive properties about sensor systems. Software agents can query the sensor instance data to determine the capabilities of connected sensor systems. Once the capabilities of the sensor systems have been determined, other agents may task the sensor systems, for example, retrieving humidity data for a specified time period (Figure 5(b)) [2–6].

### 1.4. Profiling Sensor Systems and Algorithms

**Figure 6.**
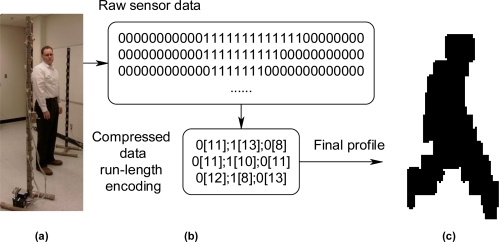
**(a)** Near-IR PFx sensor with detectors vertically deployed. **(b)** Output from an algorithm that formats PFx sensor data. **(c)** Output from an algorithm that produces a silhouette from formatted PFx data.

**Figure 7.**
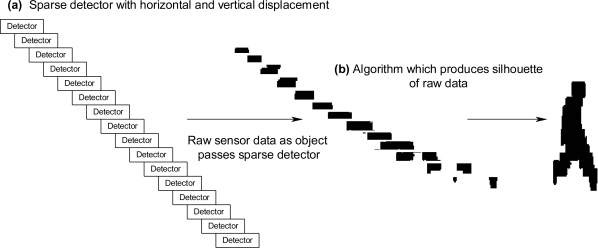
**(a)** PFx sensor with detectors deployed vertically with a horizontal displacement. **(b)** PFx raw data formatted by an algorithm as a profile.

**Figure 8.**
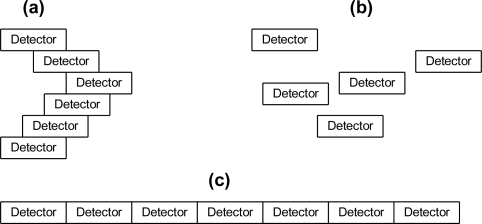
**(a)** PFx sensor with detectors deployed vertically with a specific horizontal displacement. **(b)** PFx sparse detector with random detector displacement. **(c)** PFx sparse detector with only horizontal displacement.

To show how an ontological problem-solving framework can address the challenge of matching sensor systems to compatible algorithms for a specific task, a family of unattended ground profiling sensors (denoted as PFx, in which PF_1_, PF_2_, …, PF_n_ are different types of profiling sensors) and algorithms were deployed in a prototype environment. PFx sensor systems provide unique opportunities for dynamic feature extraction through extendable algorithms and subsequent tasking. The main purpose of PFx sensors is to capture profiles of objects, which can be subsequently classified by algorithms using a variety of techniques, such as Naive Bayes algorithms, neural networks, or support vector machines. A common theme of all PFx sensors is that they are intended to be low cost and provide a profile that can be reliably classified. There are many different types of PFx sensors, which exploit various technologies, including a family of PFx imaging sensors, which use a sparse detector array. PFx sensors include, but are not limited to, novel imaging sensors in the visible, near infrared, short-wave infrared, mid-wave infrared, and long-wave infrared bands. One of the initial and simplest approaches to a PFx sensor was a prototype that used a sparse, vertical array of detectors. One configuration was on a vertical pole, as shown in Figure 6(a), while other configurations may include a horizontal displacement among the detectors as shown in Figures 7(a) and 8. Other algorithms may format or compress the raw sensor data produced by PFx sensors, as shown in Figure 6(b), or generate profiles into formats such that other algorithms can subsequently process the data, as shown in Figure 7(b). One example is a visualization algorithm, which may generate a silhouette of an object for presentation to a human evaluator for classification. Other algorithms that process PFx data may classify silhouettes as humans, animals, or vehicles [12,40–46].

**Figure 9.**
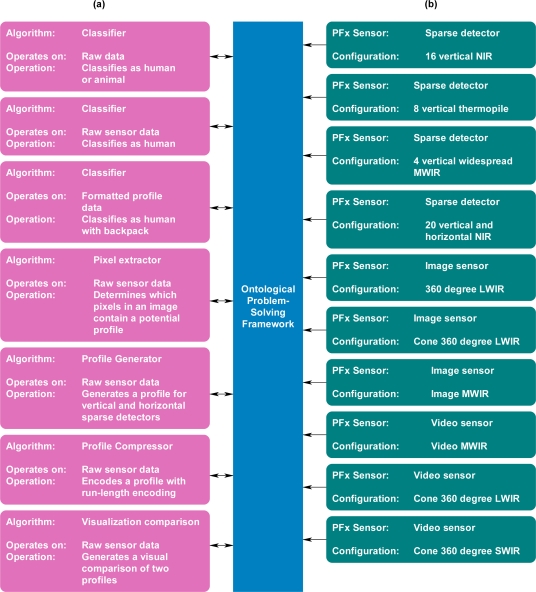
**(a)** Representative algorithm types, including classifiers, visualizers, and silhouette generators. **(b)** Representative PFx sensor types, including sparse detectors and imagers.

The PFx systems, with their various capabilities and relationships, represented a unique opportunity for integration onto the ontological problem-solving framework (Figure 9). The following section describes in detail the novel ontological problem-solving framework using PFx sensors and algorithms to illustrate the matching of sensor systems to independently designed algorithms for a task. The problem-solving approach will illustrate how PFx sensors are matched to compatible algorithms for pixel extraction, profile generation, visualization, and various other tasks. Even though the PFx sensors and algorithms are used for proof-of-principle aspects of the ontological problem-solving framework, the same approach may be extended for use by other types of sensors and algorithms to achieve different tasks.

## 2. Reasoning Process to Match Sensor Systems to Algorithms

The ontological problem-solving framework uses a reasoning process that leverages knowledge management techniques, such as semantic data modeling with ontologies, to address the challenge of matching sensors to compatible algorithms to form synthesized systems capable of satisfying a task. For this paper, the following definitions are used to describe sensors and algorithms. A sensor is a device that produces raw data while an algorithm uses the raw data for further processing These definitions are similar to ones put forth by the Open Geospatial Consortium, such as defining sensors as processes and defining sensors and algorithms as services in SensorML [34]. Of note is that low-level algorithms, which may reside on the sensor hardware, are now considered as algorithms, which are not part of the physical sensor. The low-level algorithms may be device drivers or software to process the raw sensor data into a specific format. Separating the low-level algorithms from the specific sensor systems facilitates a more flexible knowledge representation of the sensor systems and algorithms. With these definitions, meta-data, such as sensor and algorithm properties, network communications, data formats, *etc*., must be captured to explicitly represent the relationships among sensors and algorithms. The use of models to capture knowledge about sensors and algorithms facilitates inference with rules based on description logic. The knowledge models, rules, and inference engine may then allow other agents using the reasoning process of the ontological problem-solving framework to determine the capabilities of sensors and algorithms to opportunistically discover and form synthesized systems capable of satisfying a task.

### 2.1. Ontological Relationship Structure

In this work, the descriptions of algorithms and sensors are represented in an ontology similar to the approach taken with OntoSensor and CIEDETS, which were developed by knowledge engineers with input from subject sensor matter experts. Using OntoSensor and CIEDETS ontologies as a baseline for the ontological reasoning process, the ontology needed to be extended to allow for the matching of sensors to algorithms to form synthesized systems capable of satisfying a task. The baseline ontology was extended with the following: (1) a class hierarchy for describing algorithms with descriptive properties; (2) additional properties in the sensor class for describing PFx sensors; (3) an additional class hierarchy for matching sensors to compatible algorithms; and (4) additional declarative rules.

The challenge is to match sensor systems to algorithms to form synthesized systems capable of satisfying a task and then reusing those systems for other tasks. The baseline ontologies already describe sensor systems and various properties of those systems. Since the focus of the ontological problem-solving framework was to use a persistence surveillance sensing environment, properties were added to the sensor classes that describe PFx sensor systems. Generally these systems have properties, such as image resolution, geo-locations of detectors that make up a sparse detector array, and network communications. In order for a PFx sensor system to be described and represented by the ontology, these properties and others were added to various subclasses of the *Sensor* class. Algorithms were not represented by the baseline ontologies so a complete class hierarchy was added along with various attributes, such as data input/output requirements, process capabilities and purposes, descriptions of data, and network communications mapped into many different properties.

**Figure 10.**
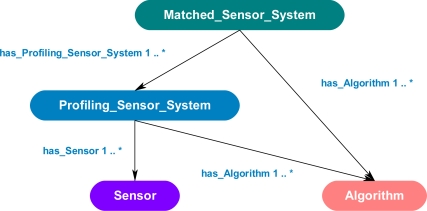
Core ontology of the ontological problem-solving framework that describes the relations of the classes: *Matched_Sensor_System*, *Profiling_Sensor_System*, *Sensor* and *Algorithm*.

If sensor systems and algorithms are matched to perform a task, the ontology must have a way to describe this possible interoperability. This combination is not merely just a sensor and a compatible algorithm, but a combination of systems that may satisfy a given task. To describe this possible combination of systems, the concept of a synthesized system was developed and integrated into the ontology. A synthesized system is a possible combination of a sensor and compatible algorithm that may satisfy a task. When looking at various types of sensor and algorithm combinations in a persistence surveillance environment, generally, a sensor creates raw data of a passing object, a profile of the passing object is created from the raw data, and then the profile has a process applied to it, such as a classification or visualization. This is a two-step process of first generating a profile and second to process this profile. This two-step process can be represented by two different synthesized systems. The first synthesized system matches a sensor to an algorithm for the task of generating profiles, while the second synthesized system is a matching of the first synthesized system to another algorithm, which has the task of processing the profile for some purpose. To represent the two types of synthesized systems in the ontology, two new classes were created that have object type properties that establish relationships back to established classes and properties. Figure 10 shows the core ontology for matching sensors to compatible algorithms to form synthesized systems, which are capable of satisfying a task, which is made up of four main classes: *Matched_Sensor_System*, *Profiling_Sensor_System*, *Sensor* and *Algorithm*. A bottom-up approach will be used to explain the purpose of each of the classes, their corresponding relationships, and the following section will describe the rules used to query the ontology instance data for possible synthesized systems.

The *Sensor* class describes a sensing device, which generates raw data. The *Algorithm* class describes a process, which requires raw sensor data or data provided by another algorithm as input and then generates output. The *Algorithm* class can include, but is not limited to, PFx data formatters, PFx classifiers, and PFx visualizers. The *Profiling_Sensor_System* class is the first synthesized system concept that describes a possible combination of a *Sensor* instance and *Algorithm* instance, which produces a profile of an object in the sensor’s field of view. The *Sensor* and *Algorithm* instances are linked to a *Profiling_Sensor_System* instance through the two object type properties called has_Sensor and has_Algorithm. A *Profiling_Sensor_System* may have many *Algorithm* instances processing the sensor data. For example, one algorithm may extract specific pixels from a raw image while another algorithm generates a profile of the extracted pixels, thus, a chain of algorithms and sensors may be matched in a *Profiling_Sensor_System*. The *Matched_Sensor_System* class is the second synthesized system concept that describes a possible combination of a *Profiling_Sensor_System* instance and *Algorithm* instance, which produces a result, such as a visualization or classification of the profile. The instances *Profiling_Sensor_System* and *Algorithm* are linked to a *Matched_Sensor_System* instance through the object type property has_Profiling_Sensor_System and has_Algorithm. A *Matched_Sensor_System* may have many algorithms processing the profile from the *Profiling_Sensor_System* instance. For example, one algorithm may convert the profile to a new format, while another algorithm operates on the new profile to generate a classification.

**Figure 11.**
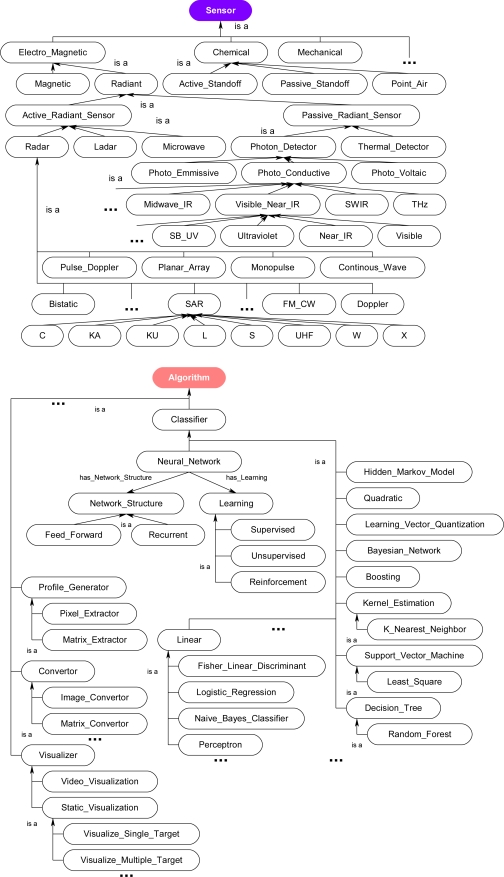
Extended class hierarchy of the ontological problem-solving framework for the *Sensor* and *Algorithm* classes.

**Figure 12.**
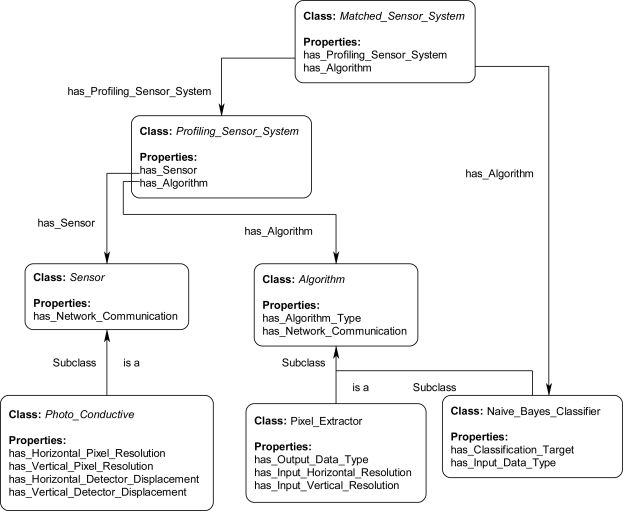
Excerpt of the properties for representative classes and subclasses for the reasoning process in the ontological problem-solving framework.

Figure 11 shows the class hierarchy of the *Sensor* and *Algorithm* classes. Each of these classes may have many properties, which are used to describe the instances. Figure 12 shows several of the properties used to describe some of the classes within the ontology. For example, the subclass *Photo_Conductive* of the *Sensor* class has specific properties describing a sensor's pixel resolution: has_Horizontal_Pixel_Resolution and has_Vertical_Pixel_Resolution while also inheriting the *Sensor* class property has_Network_Communication. The subclass *Pixel_Extractor* of class *Algorithm* has properties describing the resolution of a generated profile: has_Input_Horizontal_Resolution and has_Input_Vertical_Resolution while also inheriting the property has_Network_Communication from the *Algorithm* class. Similar in nature is the subclass Naive_Bayes_Classifier which inherits from the same Algorithm class but also adds its own unique properties such as has_Classification_Target. The *Profiling_Sensor_System* and *Matched_Sensor_System* classes also have properties, which are derived from the *Sensor* and *Algorithm* classes through rules executed during the inference process. These object and data type properties are only a few of the many describe in the ontology.

### 2.2. Ontological Rules

**Figure 13.**
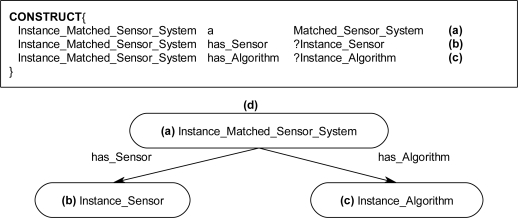
SPARQL CONSTRUCT clause **(a)** Returned *Matched_Sensor_System* instance, Instance_Matched_Sensor_System, linked to *Sensor* and *Algorithm* instances. **(b)** Instance_Sensor and **(c)** Instance_Algorithm variables instantiated to specific *Sensor* and *Algorithm* instances in the WHERE clause, thereby establishing a link between a matched *Sensor* instance and an *Algorithm* instance. **(d)** Instance diagram.

**Figure 14.**
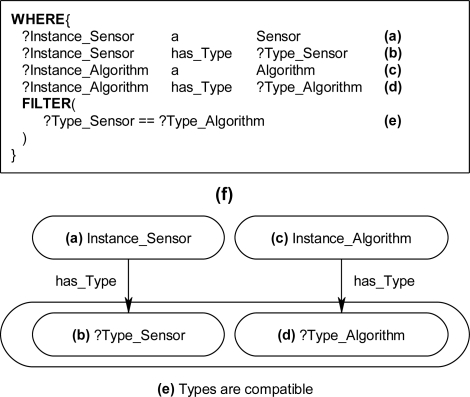
SPARQL WHERE clause **(a)** The variable Instance_Sensor instantiated to an instance of the class *Sensor* with the data property **(b)** has_Type established to the variable Type_Sensor. **(c)** The variable Instance_Algorithm instantiated to an instance of the class *Algorithm* with the data property **(d)** has_Type established to the variable Type_Algorithm. **(e)** FILTER command comparing Type_Sensor and Type_Algorithm variables for compatibility. **(f)** Instance diagram.

The graph-matching query language SPARQL [8] was used to create declarative rules for the ontological problem-solving framework. The SPARQL query language has internal functions that will allow for the querying of possible synthesized systems through an inference engine. Once the synthesized systems are returned back from the inference engine the systems can be formed into new instance data to be leveraged by other systems on the ontological problem-solving framework. The rules contain statements that consist of logical constraints among instance data and properties that must be true for subsequent instances and properties to be derived and returned as results back to the ontology. The rules are made up of two components, referred to as the WHERE and CONSTRUCT clauses. The CONSTRUCT (Figure 13(a)) clause is used to return possible object instances and properties based on instance data and properties that satisfy the WHERE clause of the SPARQL rule. The returned instances may include links to established instances (Figure 13(b,c)), as well as links to derived attributes of the returned instances. The WHERE clause contains the logical constraint statements that queried existing instances must satisfy before the CONSTRUCT clause returns the possible instances and establishes links to the pre-existing instances and properties (Figure 14). The WHERE clause constraint statements include preconditions (properties that must exist), and the other descriptive logical constraints, such as FILTER and OPTIONAL statements, that existing queried instances must satisfy before possible instances and properties are returned by the CONSTRUCT clause. Each rule can be regarded as a Horn clause in that each condition is specified in the rule via logical conjunction (logical AND). If all the properties hold true then the specified instance is returned by the rule. Logical disjunction (*i.e*., logical OR) can be regarded as a collection of rules that create a similar instance, for example, a collection of rules that each bind on different properties which return instances of a *Profiling_Sensor_System.*

**Figure 15.**
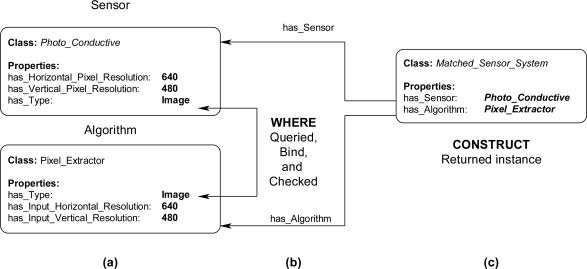
Instance diagram of a SPARQL query binding on specific instance data and returning possible instances **(a)** Existing *Sensor* and *Algorithm* instances that have has_Type values equal to “Image” **(b)** WHERE clause binding and checking the has_Type property **(c)** CONSTRUCT clause returning a possible *Matched_Sensor_System* with established links to the found *Sensor* and *Algorithm* instances.

The inference engine will process the SPARQL rules for all combinations of pre-existing instances. For example, in Figure 14(a,c), these two statements result in the WHERE clause cycling through all *Sensor* and *Algorithm* instances. The statements in Figure 14(b,d) bind the property has_Type value for the instances. The FILTER statement in Figure 14(e) compares the value of has_Type for the *Sensor* and *Algorithm* instances. If the FILTER statement is satisfied, then, the CONSTRUCT clause is subsequently executed to return the specified instance and associated properties. For a simple example, the instance data in Figure 15 will be queried with a complete SPARQL rule with the CONSTRUCT and WHERE clauses in Figures 13 and 14. The *Photo_Conductive* sensor instance and *Pixel_Extractor* algorithm instance each have the property has_Type with a value of “Image” (Figure 15(a)). When the complete SPARQL rule of Figures 13 and 14 is executed by the inference engine the WHERE clause will query for a possible *Sensor* and *Algorithm* instances whose property has_Type are the same (Figure 15(b)). Once a possible combination has been found (*Photo_Conductive* and *Pixel_Extractor* in this case), the CONSTRUCT clause will be execute by the inference engine to return the possible *Matched_Sensor_System* instance with links back to the original *Photo_Conductive* and *Pixel_Extractor* instances (Figure 15(c)). The returned *Matched_Sensor_System* instance will then be placed into the ontology for further inference and use by other systems. Even though this is a simple example with SPARQL, with additional constructs, such as the FILTER or OPTIONAL commands, far more complex rules may be built.

**Figure 16.**
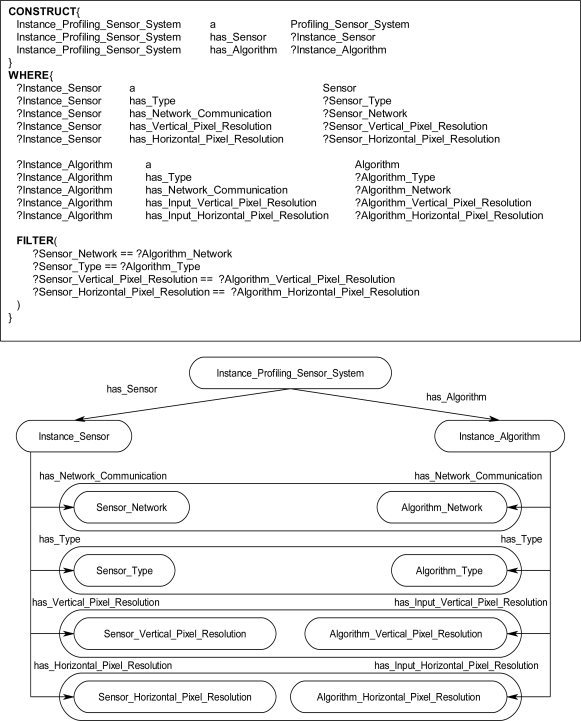
Sample rule and instance diagram. The rule returns an instance of a *Profiling_Sensor_System* if the *Algorithm* instance and *Sensor* instance are type compatible with respect to the network communication and pixel resolutions properties.

**Figure 17.**
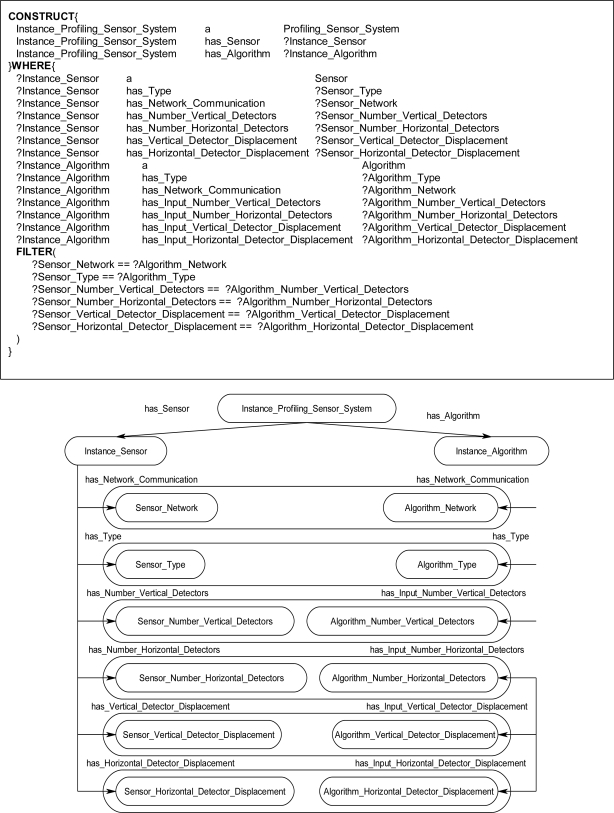
Sample *Profiling_Sensor_System* rule and instance diagram. The rule returns an instance if the *Algorithm* instance and *Sensor* instance properties: type, network communication, number of detectors, and displacement properties are compatible.

**Figure 18.**
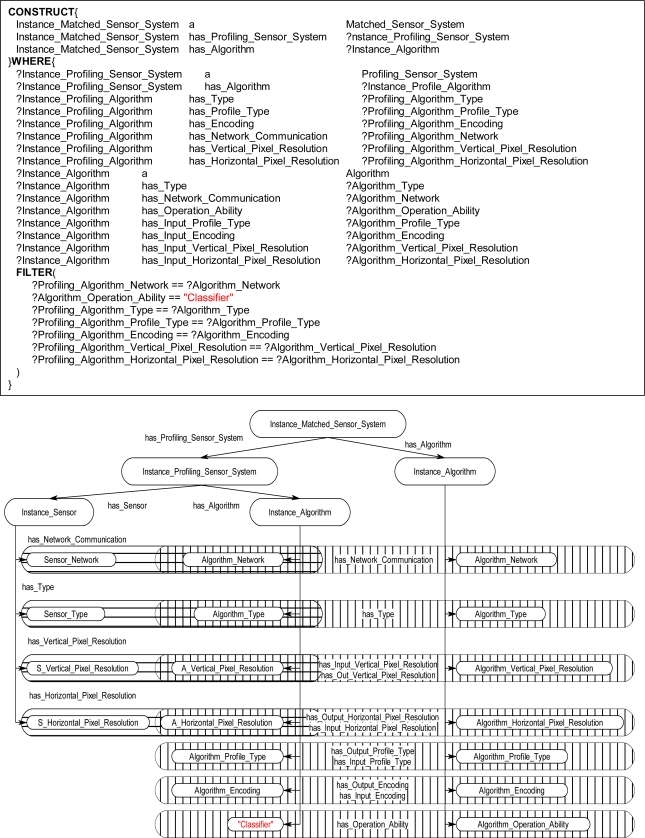
Sample *Matched_Sensor_System* rule and instance diagram. The rule returns an instance if the *Profiling_Sensor_System* instance and *Algorithm* instance properties: network communication, types, encoding, classification, and pixel resolutions are compatible.

**Figure 19.**
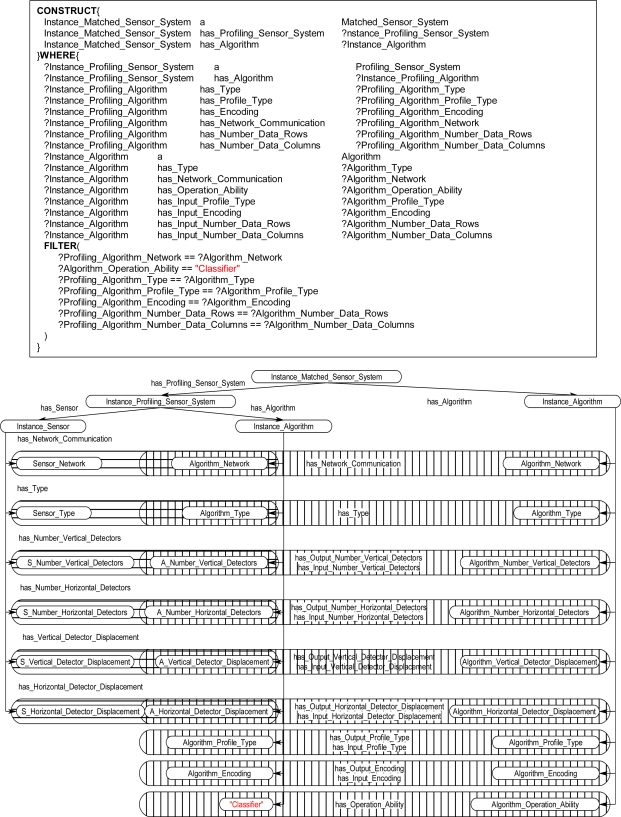
Sample *Matched_Sensor_System* rule and instance diagram, which returns an instance if the *Profiling_Sensor_System* instance and *Algorithm* instance properties: network communication, types, encoding, classification, data rows, and columns properties are compatible.

The rules in the ontological problem-solving framework bind on all combinations of *Sensor* and *Algorithm* instances. Their respective properties are then compared in the FILTER statements of the WHERE clause to determine which instances need to be returned and when to establish links between other instances. Figure 16 through Figure 19 each show one of many rules used to return possible *Profiling_Sensor_System* instances and *Matched_Sensor_System* instances. The WHERE clause in the *Profiling_Sensor_System* rules in Figures 16 and 17 bind on the properties of *Sensor* and *Algorithm* instances, such as pixel resolution in Figure 16, number of detectors in Figure 17, and type for both Figures 16 and 17. Further, in the WHERE clause, the FILTER statement now compares specific *Sensor* instance properties to the *Algorithm* instance properties. For example, in Figure 16, the FILTER statement compares the network communication type and pixel resolutions. Once a set of instances for a *Sensor* and *Algorithm* have been queried, which satisfy the constraints of the WHERE clause, the CONSTRUCT clause will then return a *Profiling_Sensor_System* instance and establish links to the compatible *Sensor* and *Algorithm* instances. The same process occurs in the WHERE clause in Figure 17, but instead of comparing pixel resolutions, detector properties are compared for compatibility. The rules for *Matched_Sensor_System* in Figures 18 and 19 follow a similar logical process as the *Profiling_Sensor_System* rule. The only difference between the rules, other than the specific properties of the instances, is in the FILTER statement where an additional statement constrains the WHERE clause to a specific type of *Algorithm*, in this case a “Classifier”. The rules shown in Figures 14 and 19 both return *Matched_Sensor_System* instances, which will classify the generated profiles of *Profiling_Sensor_System* instances.

### 2.3. Instances of Profiling Sensor Systems and Algorithms on Ontological Problem-Solving Framework

**Figure 20.**
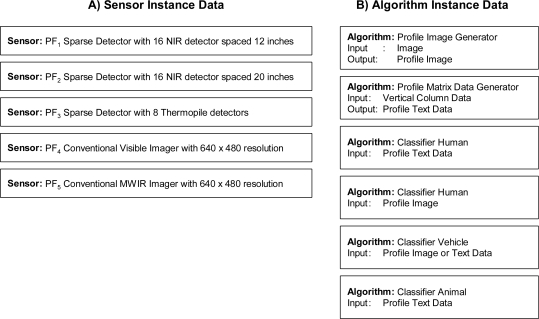
Example instances: **(a)** Three PFx sensors and two conventional imaging sensors. **(b)** Two profile generators and four classifiers.

**Figure 21.**
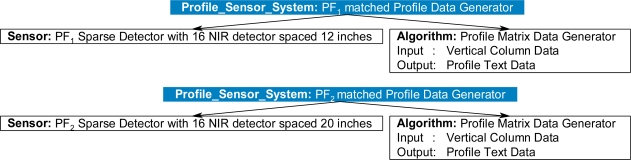

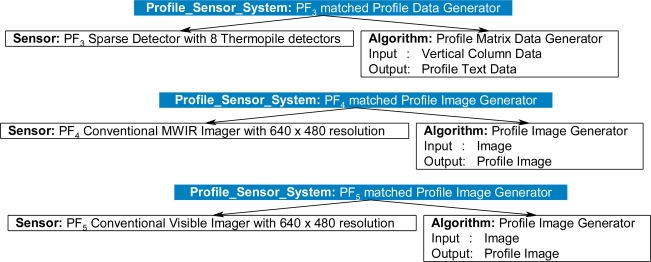
Five new *Profiling_Sensor_System* instances returned, with derived relationships, after the first pass of the inference cycle.

To illustrate a simple case, Figure 20 shows five sensor instances, including three PFx sensors and two conventional imagers, and six algorithms, including two profile generators and four different classifiers, with different property specifications and requirements. When the inference cycle begins, the rules from Figure 16 through Figure 19 will execute. On the first pass of the inference cycle, five new *Profiling_Sensor_System* instances were created, as shown in Figure 21. The two algorithms Profile Image Generator and Profile Matrix Data Generator were matched to multiple sensors based on constraints of the algorithms and specifications of the sensors. For example, the *Algorithm* instance Profile Image Generator was matched to the *Sensor* instance PF_5_ Conventional Visible Imager because the constraint of requiring image data for the Profile Image Generator was satisfied.

**Figure 22.**
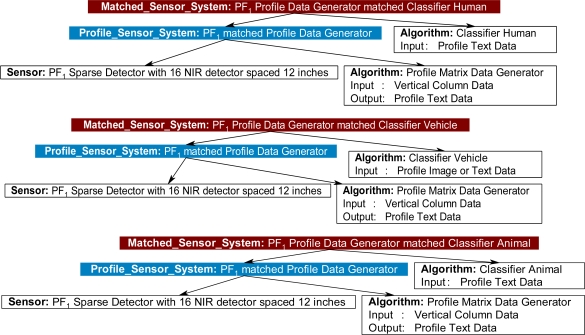

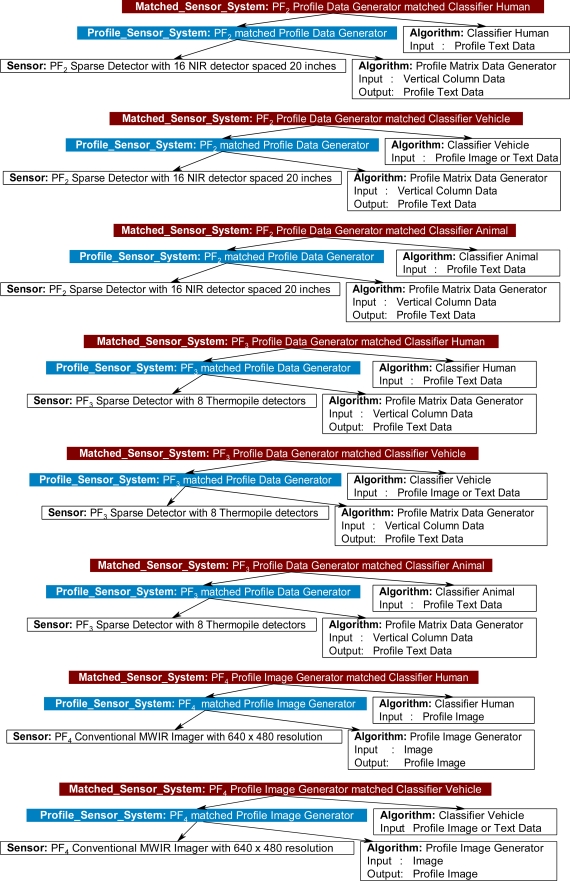

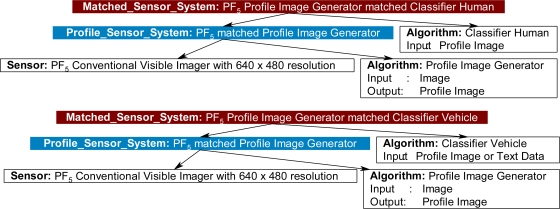
Thirteen new *Matched_Sensor_System* instances returned, with derived relationships, after the second pass of the inference cycle.

During the second pass of the inference cycle, thirteen new *Matched_Sensor_System* instances were created, as shown in Figure 22. The four different classifiers were matched to multiple *Profiling_Sensor_System* instances based on the type of profile generated and the requirements of the classifiers. For example, the *Profiling_Sensor_System* instance PF_1_ matched Profile Data Generator was matched to the *Algorithm* instance Human Classifier because the constraint of requiring text data was satisfied for the Human Classifier. On the third pass of the inference cycle, no new instances were created; therefore, the inference cycle halts and returns all matches.

## 3. Discussion

The challenge was to match sensor systems to compatible algorithms to form synthesized systems, which are capable of satisfying a task and matching those systems to new systems for other tasks. The sample rules described in this paper specified relatively simple compatibility constraints among sensors and algorithms. However, even with these simple rules, it is noteworthy that the *Algorithm* instances were matched to multiple *Sensor* and *Profiling_Sensor_System* instances thus achieving the ability to reuse those systems for tasks that may have not been anticipated at the time the sensors and algorithms were first deployed. For example, of the five synthesized system concept *Profiling_Sensor_System* instances that were returned, the algorithm Profile Matrix Generator was matched to three different sensor systems and the algorithm Profile Image Generator was matched to two sensor systems. If not for the matching and return of the *Profiling_Sensor_System* synthesized systems, each one of the matched systems would have had to be individually designed.

The same results can be seen in the synthesized system *Matched_Sensor_System,* which reused the five *Profiling_Sensor_System* synthesized systems in thirteen systems with different tasks, such as visualizing or classifying the profiles. If the original algorithms represented by the *Algorithm* instances had been designed for specific *Sensor* instances, the reasoning process of the ontological problem-solving framework would not have matched the algorithms to new sensors, thus the sensor systems and algorithms would have had to be re-engineered specifically for one another to satisfy a task. It is important to note that the synthesized system concepts *Profiling_Sensor_System* and *Matched_Sensor_System* capture more than just a *Sensor* matched to an *Algorithm*. The concept synthesized systems, represent new systems which are capable of performing a task. Other rules in the ontological problem-solving framework may operate on far more than just two attributes for establishing interoperability via matching constraints. The rules may determine that multiple matched *Profiling_Sensor_System* and *Matched_Sensor_System* instances may be formed into new more complex synthesized systems, which may be capable of satisfying more complex tasks, which may include statistical analysis on multiple profiles. With the formation of the synthesized system by the reasoning process, the ontological problem-solving framework may create more complex synthesized systems. These more complex systems may then be assigned to subtasks of high-level missions by other systems on the network coordinating and executing the mission. Without the use of the ontology, rules, and inference engine these sensors and algorithms would have had to be designed *a priori* as a synthesized system for every new task. However, many of these new tasks are not known at the time the systems are deployed; therefore, opportunistically discovering compatible systems and dynamically creating matched synthesized systems which are capable of satisfying a new task through inference is important.

Currently, the reasoning process of the ontological problem-solving framework is still in a prototype stage so scale-up performance analysis is limited. The problem-solving framework can scale to large numbers of sensors and algorithms, but the time to compute all combinations of sensors and algorithms is based on the computational complexity of the inference engine, which is influenced in part by the reasoning strategy and the expressiveness of the knowledge representation formalism. For the performance to increase, the inference engine must check multiple algorithms in parallel or the ontological problem-solving framework must invoke the inference engine multiple times in parallel with different algorithms and keep track of which instances are being checked to stop redundant bindings. Even though the ontological problem-solving framework is still in the prototype stage, performance issues and solutions are being studied; however, the logical framework is the priority at this stage of research.

## 4. Conclusions

Challenges, such as matching sensors to compatible algorithms that may satisfy a task, will become even more difficult with the continued development and deployment of new sensor systems and algorithms. Compounding the challenge is the lack of knowledge models used to explicitly capture the design and capabilities of sensor systems and algorithms. By leveraging knowledge models, sensor systems and algorithms can be matched together in real-time without the need to design those matched systems specifically for one another *a priori*, thus facilitating the use of these assets in new and innovative ways not originally anticipated on deployment. To exploit the power of knowledge models, algorithms must become less dependent on any given sensor data source, thus sensor systems and algorithms must describe their respective attributes and capabilities in a machine-interpretable format to allow the reasoning process to infer which systems may be matched together into more complex synthesized systems. The reasoning process of the ontological problem-solving framework discussed in this paper is the first step to achieving this goal and addressing the challenge of matching systems that are capable of satisfying a task. Even though the reasoning process of the ontological problem-solving framework was described in the context of profiling sensor systems and algorithms, the overall approach may be used for other types of sensor systems and algorithms to form different types of synthesized systems capable of satisfying new tasks.
